# A machine-learning algorithm using claims data to identify patients with homozygous familial hypercholesterolemia

**DOI:** 10.1038/s41598-024-58719-y

**Published:** 2024-04-17

**Authors:** Jing Gu, Matthew Epland, Xinshuo Ma, Jina Park, Robert J. Sanchez, Ying Li

**Affiliations:** 1 Regeneron Pharmaceuticals, Inc., 777 Old Saw Mill River Road, Tarrytown, New York, NY 10591 USA; 2 Komodo Health, New York, NY USA

**Keywords:** Dyslipidaemias, Cardiology, Diseases, Medical research

## Abstract

Homozygous familial hypercholesterolemia (HoFH) is an underdiagnosed and undertreated ultra-rare disease. We utilized claims data from the Komodo Healthcare Map database to develop a machine-learning model to identify potential HoFH patients. We tokenized patients enrolled in MyRARE (patient support program for those prescribed evinacumab-dgnb in the United States) and linked them with their Komodo claims. A true positive HoFH cohort (n = 331) was formed by including patients from MyRARE and patients with prescriptions for evinacumab-dgnb or lomitapide. The negative cohort (n = 1423) comprised patients with or at risk for cardiovascular disease. We divided the cohort into an 80% training and 20% testing set. Overall, 10,616 candidate features were investigated; 87 were selected due to clinical relevance and importance on prediction performance. Different machine-learning algorithms were explored, with fast interpretable greedy-tree sums selected as the final machine-learning tool. This selection was based on its satisfactory performance and its easily interpretable nature. The model identified four useful features and yielded precision (positive predicted value) of 0.98, recall (sensitivity) of 0.88, area under the receiver operating characteristic curve of 0.98, and accuracy of 0.97. The model performed well in identifying HoFH patients in the testing set, providing a useful tool to facilitate HoFH screening and diagnosis via healthcare claims data.

## Introduction

Homozygous familial hypercholesterolemia (HoFH) is a rare autosomal dominant genetic disorder of lipid metabolism, characterized by markedly elevated circulating levels of low-density lipoprotein cholesterol (LDL-C)^1,2^. Exposure to chronically elevated LDL-C levels from birth accelerates the development of atherosclerotic cardiovascular disease (ASCVD), and increases the risk of adverse cardiovascular events and premature mortality^1,3,4^.

HoFH is caused by mutations in genes that encode for proteins involved in the hepatocellular uptake and catabolism of low-density lipoprotein (LDL)^1,5^. Loss-of-function mutations in the low-density lipoprotein receptor (*LDLR*) gene account for approximately 90% of genetically confirmed HoFH cases^6^. Less often, HoFH arises due to mutations in the apolipoprotein B (*APOB*), proprotein convertase subtilisin/kexin type 9 (*PCSK9*), and LDL protein receptor adaptor 1 (*LDLRAP1*) genes^6^.

HoFH has an estimated global prevalence of 1 in 250,000^7,8^. However, the true prevalence of HoFH is unknown, as the condition remains systematically underdiagnosed and undertreated^2^. A recent analysis of the Family Heart Database, which includes more than 81 million Americans, identified 277 patients with a lipid profile similar to patients with genetically confirmed HoFH^2^. Despite a median LDL-C of 444 mg/dL for these 277 patients, only 25.6% had a diagnosis of familial hypercholesterolemia (FH), 39.7% were not receiving any lipid-lowering therapies (LLTs), and 18.8% were on high-intensity statins alone^2^.

Patients with HoFH usually present in childhood with cutaneous xanthomas (extravascular cholesterol deposition) in the tendons and joints, which may be the first visible disease symptoms^6^. Cholesterol depositions may also appear on the eyelids (xanthelasma) and corneal arcus in the eyes^6^. However, not all patients with HoFH present with these pathognomonic physical findings.

If patients remain untreated, LDL-C levels may rise above 400 mg/dL, four times the age- and gender-related mean level, and most patients will develop overt atherosclerosis before the age of 20 years^1,6^. This can manifest as early coronary artery disease and valvular heart disease, resulting in a high risk for myocardial infarction, stroke, or sudden death in the first or second decade of life^1,4,9,10^. Patients with HoFH either do not respond to traditional LLTs (for example, statins, ezetimibe, and proprotein convertase subtilisin/kexin type 9 [PCSK9] inhibitors) or have attenuated responses, as the effectiveness of these LLTs is dependent upon functioning LDLRs^11–14^.

Given the low awareness and diagnosis rate, as well as the severe outcomes of this disease, it is essential to improve screening and identification of HoFH to facilitate early diagnosis and timely treatment. However, the heterogeneity of phenotypes and lack of an International Classification of Diseases (ICD)-10 diagnosis code make it challenging to identify patients with HoFH^5,15^.

Despite the phenotypic heterogeneity of HoFH, clinical features such as abnormally elevated levels of LDL-C, use of multiple LLTs, clinical signs in early life, and early-onset cardiovascular disease could act as identifying factors. Administrative medical claims data provide a rich historical record of these and many other clinical features. Machine-learning models trained on administrative medical claims data present a unique approach to disease diagnosis. Thus, the aim of our study was to use administrative claims data to develop a machine-learning model that could potentially identify patients with HoFH.

## Methods

### Data sources

In this retrospective observational study, we used de-identified patient-level claims data from the Komodo Healthcare Map database (October 1, 2015, to March 31, 2022) to build and train a high-performance machine-learning model for the potential identification of patients with HoFH. The Komodo Healthcare Map database encompasses longitudinal, medical, and prescription claims data on more than 325 million insured patients who are well-distributed geographically across the United States^16^. The database is representative of the general United States population when compared with the Centers for Disease Control and Prevention National Health Interview Survey^17^.

As there is no ICD-10 diagnosis code available for HoFH^18^, patients with HoFH could not be directly identified from the Komodo Healthcare Map database. Instead, patients with HoFH were identified using (1) prescription claims for lomitapide and evinacumab-dgnb from the Komodo Healthcare Map database, and (2) inclusion in the MyRARE program. MyRARE is a patient support program designed to provide financial support and resources for patients who have been prescribed with evinacumab-dgnb in the United States. Evinacumab is an angiopoietin-like 3 inhibitor, approved for the treatment of HoFH as an adjunct to other LLTs in those aged 5 years and older^19^. All patients enrolled in the MyRARE program have a clinically or genetically confirmed diagnosis of HoFH.

Patients enrolled in MyRARE were de-identified and tokenized using Datavant (Datavant, Inc., San Francisco, CA), a privacy-preserving record-linkage software. The tokens generated for each patient were then used to link patients with their Komodo claims data. When applicable, the data source for LDL-C values in the past 7 years was from Quest Diagnostics (Secaucus, NJ), which is linked to the Komodo Healthcare Map database.

Informed consent was obtained from MyRARE patients to use their de-identified information for conducting research, including linkage with other de-identified information from other sources. As all linked database records were de-identified and fully complied with US patient confidentiality requirements set forth in Sections 164.514 (a)–(b) of the Health Insurance Portability and Accountability Act regarding the determination and documentation of statistically de-identified data^20^, institutional review board approval was not required.

### Modeling strategy

Diagnosis and treatment information data from the Komodo Healthcare Map database and the MyRARE program was used to form a true positive HoFH cohort and a negative cohort. A machine-learning model was subsequently constructed and then trained to differentiate the positive cohort from the negative cohort. The index date on which the machine learning model was applied corresponds to the final claims made by patients in this cohort.

The true positive HoFH cohort was formed by including patients enrolled in the MyRARE program or patients with at least one prescription claim for lomitapide after January 1, 2018, or with at least one prescription claim for evinacumab-dgnb after February 11, 2021. The negative cohort was formed using patients with at least one cardiovascular disease or who were at high risk for cardiovascular disease, i.e., with a diagnosis eligible for primary prevention such as hypertension, hypercholesterolemia, hyperlipidemia, diabetes, or chronic kidney disease and who did not meet the inclusion criteria for the positive cohort. Each patient in the true positive HoFH cohort was matched to five patients in the negative cohort using stratified sampling. Simple random sampling was run within strata including gender, age groups, deciles of volume of medical claims, and pharmacy claims. Patients from both cohorts were randomly divided into training and testing sets, at a ratio of 80% to 20%, respectively, to tune the parameters and measure model performance.

Candidate features were extracted for each patient in the positive and negative cohorts, to form the basis for testing and evaluation in the machine-learning models. These candidate features were selected based on input from clinical experts following an investigation of all the information available in the claims data, including demographics, diagnoses, procedures, laboratory orders, medications, visits, and physician types. Once patient features were selected, several machine-learning models were developed using a variety of algorithms, such as logistic regression, random forest, boosted decision trees, positive-unlabeled learning, and fast interpretable greedy-tree sums (FIGS)^21^. The final adopted machine-learning algorithm was chosen based on performance scores and interpretability. With the final chosen algorithm, two machine-learning models were developed and fine-tuned: Model 1 was a completely claim-based model whereas Model 2 was a claim and laboratory value-based model. Model performance was measured using receiver operating characteristic (ROC) curves.

## Results

**Figure 1:**
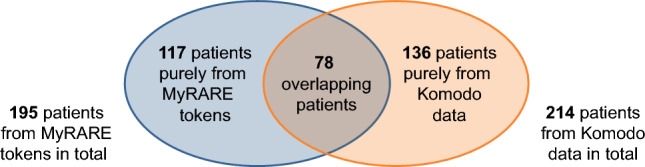
Composition of positive HoFH cohort patients. The 331 HoFH patients who comprised the positive cohort were identified from either tokens (n = 195) of patients enrolled in MyRARE, or from patients with prescriptions for lomitapide or evinacumab-dgnb from the Komodo Healthcare Map database (n = 214). *HoFH* homozygous familial hypercholesterolemia.

Overall, 201 patients enrolled in the MyRARE program were tokenized, 195 (97.0%) of whom had claims records in the Komodo database and served as part of the positive cohort. The positive cohort was additionally augmented with 136 patients who had evinacumab-dgnb or lomitapide claims in the Komodo database. This resulted in a total of 331 patients with HoFH in the positive cohort (Fig. 1). For the negative cohort, we identified 181,850,576 patients in the Komodo database with cardiovascular disease or who were at risk for cardiovascular disease.

Patient characteristics for the true positive HoFH cohort and the negative cohort are presented in Table 1. In the true positive cohort, the mean age of patients was 56 years, 55.9% were female, and 66.2% had a formal diagnosis of FH. The most common comorbidities among the positive cohort were hyperlipidemia (89.7%), acute ischemic heart disease (79.5%), and hypertension (71.9%). In the negative cohort, the mean age of patients was 65 years, 52.7% were female, and 0.8% had a formal diagnosis of FH. Among the negative cohort, hypertension (68.2%), hyperlipidemia (61.2%), and acute ischemic heart disease (48.1%) were identified as the most common comorbidities. LLT use was substantially higher among patients in the positive versus negative cohort (high-intensity stains: 68.0% vs 26.5%, ezetimibe: 54.1% vs 2.2%, PCSK9 inhibitors: 58.9% vs 0.3%, lipoprotein apheresis: 15.7% vs 0%).

**Table 1:** Patient characteristics for the true positive HoFH cohort and the negative cohort.

Characteristic	True positive HoFH cohort (n = 331)	Negative cohort (n = 181,850,567)
Age, mean, years	56	65
Female, n (%)	185 (55.9)	95,907,223 (52.7)
Medical history, n (%)		
Acute ischemic heart disease	263 (79.5)	87,406,195 (48.1)
Acute myocardial infarction	57 (17.2)	7,321,749 (4.0)
Coronary revascularization	31 (9.4)	1,336,159 (0.7)
Cardiac arrest	28 (8.5)	6,731,065 (3.7)
Chronic ischemic heart disease	162 (48.9)	31,945,672 (17.6)
Chronic kidney disease	59 (17.8)	24,602,214 (13.5)
Diabetes	104 (31.4)	53,185,027 (29.2)
Heart failure	67 (20.2)	20,864,184 (11.5)
Hyperlipidemia	297 (89.7)	111,270,683 (61.2)
Hypertension	238 (71.9)	124,112,961 (68.2)
Ischemic cerebrovascular disease	93 (28.1)	12,488,146 (6.9)
Ischemic stroke	31 (9.4)	10,729,805 (5.9)
Peripheral artery disease	109 (32.9)	24,667,296 (13.6)
Familial hypercholesterolemia	219 (66.2)	1,371,142 (0.8)
Cornea arcus	16 (4.8)	713,452 (0.4)
Lipid-lowering therapy, n (%)		
High-dose statin	225 (68.0)	48,103,959 (26.5)
Low-dose statin	58 (17.5)	23,441,052 (12.9)
Ezetimibe	179 (54.1)	4,053,067 (2.2)
PCSK9 inhibitor	195 (58.9)	620,850 (0.3)
Lipoprotein apheresis	52 (15.7)	1688 (0.0)

*HoFH* homozygous familial hypercholesterolemia, *PCSK9* proprotein convertase subtilisin/kexin type 9.

Stratified sampling was used to build a matched negative cohort, whereby each patient in the positive cohort was matched to five patients in the negative cohort. The matched negative cohort comprised 1423 patients. Thus, a total of 1754 patients (positive and negative cohorts) were included in this analysis. In total, 10,616 features of these patients were investigated, from which 87 were selected due to their clinical relevance and impact on prediction performance (Table S1). Of the different machine-learning algorithms that were explored, FIGS was selected as the final machine-learning tool based on both performance and interpretability (Table S2), and was further fine-tuned.

**Figure 2:**
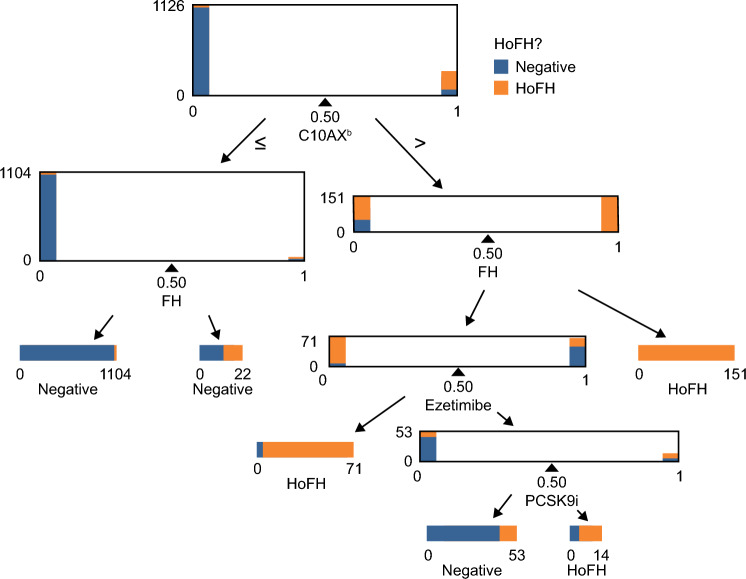
Features included in claims-based FIGS Model 1^a^. ^a^All features were used in the form of onset age. Patients never experiencing a condition were treated as having an age of onset of 90 years. ^b^C10AX is the Anatomical Therapeutic Chemical classification code for other lipid-lowering therapies. The C10AX feature was constructed excluding evinacumab-dgnb and lomitapide to avoid target leakage. *C10AX* Anatomical Therapeutic Chemical classification code for other lipid-lowering therapies, *FH* familial hypercholesterolemia, *FIGS* fast interpretable greedy-tree sums, *HoFH* homozygous familial hypercholesterolemia, *PCSK9i* proprotein convertase subtilisin/kexin type 9 inhibitor.

**Figure 3:**
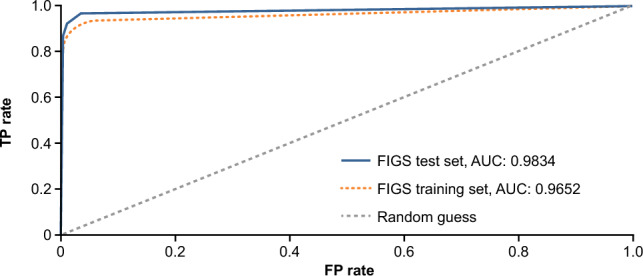
Results from the claims-based FIGS Model 1. *AUC* area under the curve, *FIGS* fast interpretable greedy-tree sums, *FP* false positive, *TP* true positive.

In the final claims-based FIGS Model 1, four useful features (ezetimibe, PCSK9 inhibitors, other LLTs [Anatomical Therapeutic Chemical classification code: C10AX], and FH) were included (Fig. 2). In the test set, the model yielded precision (positive predicted value) of 0.98, recall (sensitivity) of 0.88, area under the ROC curve of 0.98, and accuracy of 0.97 (Table 2; Fig. 3). Advanced LLTs and diagnosis of FH appeared to be the most important features included in this model.

**Table 2:** Results from claims-based FIGS Model 1 in the test set.

	True class		Measures
	Positive	Negative	
Predicted class			
Positive	60 (TP)	1 (FP)	PPV (precision): 0.98
Negative	8 (FN)	271 (TN)	NPV: 0.97
Measures	Sensitivity (recall): 0.88	Specificity: 0.99	Accuracy: 0.97

*FIGS* fast interpretable greedy-tree sums, *FN* false negative, *FP* false positive, *NPV* negative predictive value, *PPV* positive predictive value, *TN* true negative, *TP* true positive.

**Figure 4:**
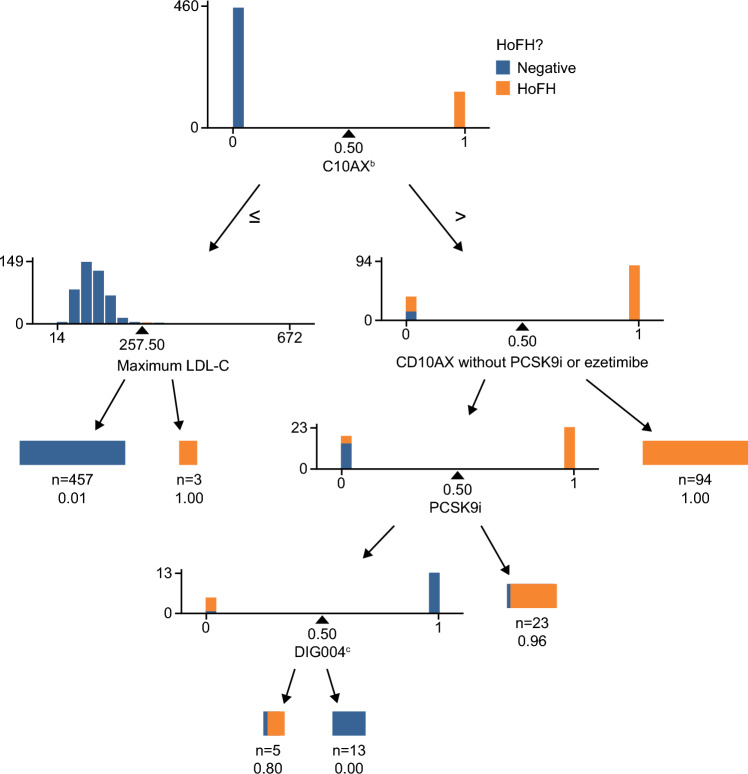
Features included in claims and laboratory values-based FIGS Model 2^a^. ^a^All features were used in the form of onset age. Patients never experiencing a condition were treated as having an age of onset of 90 years. ^b^C10AX is the Anatomical Therapeutic Chemical classification code for other lipid-lowering therapies. The C10AX feature was constructed excluding evinacumab-dgnb and lomitapide to avoid target leakage. ^c^Clinical Classifications Software Refined category for esophageal disorders. *C10AX* Anatomical Therapeutic Chemical classification code for other lipid-lowering therapies, *DIG004* Clinical Classifications Software Refined category for esophageal disorders, *FIGS* fast interpretable greedy-tree sums, *HoFH* homozygous familial hypercholesterolemia, *LDL-C* low-density lipoprotein cholesterol, *PCSK9i* proprotein convertase subtilisin/kexin type 9 inhibitor.

**Figure 5:**
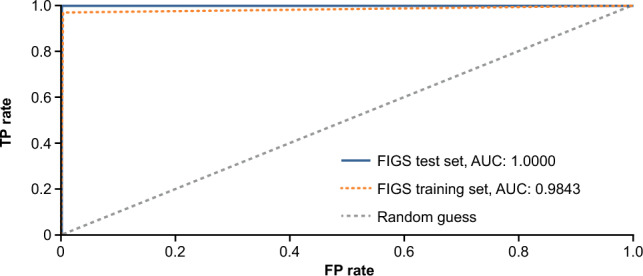
Results from the claims and laboratory values-based FIGS Model 2. *AUC* area under the curve, *FIGS* fast interpretable greedy-tree sums, *FP* false positive, *TP* true positive.

In the claims and laboratory values-based FIGS Model 2, laboratory values for LDL-C were also included in the analysis. Different features incorporating LDL-C values were explored, including the percentage of patients who had LDL-C > 190 mg/dL following treatment with ezetimibe or PCSK9 inhibitors, maximum LDL-C levels, and the total number of LDL-C tests (Table S3). Five useful features were included in the model (C10AX, C10AX without ezetimibe and PCSK9 inhibitor, PCSK9 inhibitors, maximum LDL-C level, and esophageal disorders [Clinical Classifications Software Refined category: DIG004]; Fig. 4). In the test set, the model yielded precision (positive predicted value) of 0.93, recall (sensitivity) of 1, area under the ROC curve of 1, and accuracy of 0.99 (Table 3; Fig. 5).

**Table 3:** Results from claims and laboratory values-based FIGS Model 2 in the test set.

	True class		Measures
	Positive	Negative	
Predicted class			
Positive	14 (TP)	1 (FP)	PPV (precision): 0.93
Negative	0 (FN)	117 (TN)	NPV: 1
Measures	Sensitivity (recall): 1	Specificity: 0.99	Accuracy: 0.99

*FIGS* fast interpretable greedy-tree sums, *FN* false negative, *FP* false positive, *NPV* negative predictive value, *PPV* positive predictive value, *TN* true negative, *TP* true positive.

## Discussion

The ability to identify meaningful patterns in large healthcare-related datasets makes the use of machine learning an attractive option for the screening and diagnosis of diseases that are underdiagnosed in the general population. Our retrospective observational study demonstrates the applicability of machine-learning models using a FIGS algorithm for the identification of patients with HoFH, using claims and laboratory values-based data.

Machine-learning approaches present an opportunity to leverage claims data to develop predictive models at scale. The success of a machine-learning model is dependent on the data and the performance of the learning algorithms trained through real-world data^22^. In the test set, both the claims-based FIGS model and the claims and laboratory values-based FIGS model demonstrated high precision and sensitivity in differentiating the positive HoFH cohort from the negative cohort. The machine-learning algorithm determined that the use of advanced LLTs and a diagnosis of FH were the most important features for identification of a patient with HoFH.

As HoFH is a rare disease, its diagnosis in clinical practice may be overlooked, which in part can be attributed to a lack of awareness among clinicians. A 2011 survey of cardiologists conducted by the American College of Cardiology revealed that fewer than 30% recognized FH when shown a National Lipid Association case example^23^. Furthermore, none of the surveyed cardiologists were aware that patients with FH are 20-fold more likely to develop early-onset coronary heart disease compared to the general population^23^. It is important to note that, while this algorithm is useful in detecting HoFH among patients treated for high cholesterol among providers who treat their patients for high lipids but who are as yet unaware of the disease, the usefulness of this algorithm to identify HoFH among patients never treated for lipids is not possible. While the use of genetic testing is increasing and is helpful to confirm a diagnosis, its widespread use is still in its infancy; coupled with the fact that the decision to use genetic tests is a personal choice, a phenotypic diagnosis of HoFH is sufficient to confirm disease.

Early detection and treatment of HoFH is essential in reducing the cumulative burden of elevated LDL-C and reducing the risk of developing early-onset ASCVD. Data from 67 patients with HoFH in the Cascade Screening for Awareness and Detection of Familial Hypercholesterolemia Registry revealed that at the time of enrollment into the registry, 78.4% of adults and 43.8% of children with HoFH already had documented coronary artery disease^2^. In most countries, including the United States, there is currently no population-based systematic way of identifying new index HoFH cases^24^. The current approach to identify individuals with FH is focused on opportunistic screening of high-risk patients, and cascade screening in families of affected individuals using LDL-C levels and/or genetic testing^25^. Applying machine-learning to large healthcare-related datasets may facilitate early screening and diagnosis, aid early initiation of LLTs, and delay the development of ASCVD. Our model predicted that 145,323 patients would have HoFH among the entire negative cohort, which includes over 181 million patients with or at high risk for ASCVD. Therefore, the model narrows down the potential population to less than 0.1%. However, we do acknowledge that the predicted number of HoFH patients is higher than expected based on the prevalence of HoFH at 1 in 250,000, which is calculated based on Hardy–Weinberg equilibrium and FH prevalence of 1 in 250^7,8^. Nevertheless, ultra-rare disease detection using machine learning is extremely challenging and our study provided valuable experience for future studies.

We recognize that this study has several limitations. The Komodo Healthcare Map database only provided data going back to 2015, and this was relied upon to calculate the likelihood of a patient developing HoFH. Some important information, especially from the early years of the patient journey, could not be used to train the machine-learning model. Due to the lack of an ICD-10 diagnosis code for HoFH, there is a reliance on patients taking evinacumab-dgnb or lomitapide to be classified as true HoFH positives. Consequently, the true positive HoFH cohort may not be representative of the entire HoFH population in the United States, as younger patients are less likely to be on these LLTs. Moreover, information about laboratory results, treatments, and diagnosis are extremely sparse for young patients in claims data. Therefore, the machine-learning algorithm may have limited capability in identifying pediatric or young patients with HoFH. As with any claims databases, we rely on administrative claims data for clinical details, which are subject to data coding limitations and data entry error. For example, while xanthomas are important symptoms for patients with HoFH, we are unable to characterize them in our cohort due to lack of specific ICD-10 codes. Furthermore, providers may not capture all diseases and conditions of the patient since these codes are used for billing purposes and not for diagnostic purposes. The proposed machine-learning model has also not been evaluated using independent claims data. A prospective assessment of the method is desirable.

## Conclusion

Our analysis demonstrates that machine-learning models constructed using claims and laboratory data performed well in identifying patients with HoFH in the test set, thereby providing a useful tool to facilitate the screening and diagnosis of HoFH. The features predictive of patients with HoFH included use of advanced LLTs and a diagnosis of FH. To confirm the generalizability of these findings, future work will include validation in other claims and clinical data sets, ideally in a prospective study.

### Supplementary Information

Supplementary Tables.

## Data Availability

The data underlying this article will be made available from the corresponding author on reasonable request.
