# Supplementary material for: A machine-learning algorithm using claims data to identify patients with homozygous familial hypercholesterolemia
Source: Sci Rep. 2024 Apr 17;14:8890. doi: 10.1038/s41598-024-58719-y (PMC11024086; doi:10.1038/s41598-024-58719-y)
Supplement: Supplementary file 1 — Supplementary Tables. [file 41598_2024_58719_MOESM1_ESM.docx]

# Supplementary Appendix

# Table S1. Features selected for assessment in the models^a^

| **Category** | **Description** | **Code count** |
| --- | --- | --- |
| Drug | C10AX^b^, PCSK9 inhibitor, ezetimibe, high-dose statin, low-dose statin, anti-diabetes, beta blockers, loop diuretics, analgesics, other cardiovascular agents, sympathomimetic agents, thiazide diuretics, proton pump inhibitors, osmotically acting laxatives, potassium, platelet aggregation inhibitors, ACE inhibitors, ARBs, bile acid sequestrants, anti-infectives, etc. | 29 |
| Disease | Familial hypercholesterolemia, family history of familial hypercholesterolemia, acute myocardial infarction, cardiac arrest, heart failure, hypercholesterolemia, ischemic stroke, PAD, unstable angina, etc. | 25 |
| HCPCS | LDL-C tests, other lipid tests, lipoprotein apheresis, dilated retinal eye exam, diagnostic radiology, magnetic resonance angiography, radiologic examination on chest, transesophageal echocardiography, anesthesia for upper GI procedures, etc. | 19 |
| HCP/HCO Specialty | Cardiology, surgery vascular, dermatology, internal medicine, ophthalmology, endocrinology, diagnostic radiology, etc. | 12 |
| Demographic | Patient age, gender | 2 |
| **Grand total** |  | **87** |

^a^All features were used in the form of onset age. Patients never experiencing a condition were treated as having an age of onset of 90 years.

^b^C10AX is the Anatomical Therapeutic Chemical classification code for other lipid-lowering therapies. The C10AX feature was constructed excluding evinacumab-dgnb and lomitapide to avoid target leakage.

ACE, angiotensin-converting enzyme; ARB, angiotensin receptor blocker; C10AX, Anatomical Therapeutic Chemical classification code for other lipid-lowering therapies; HCO, healthcare organization; HCP, healthcare professional; HCPCS, Healthcare Common Procedure Coding System; GI, gastrointestinal; LDL-C, low-density lipoprotein cholesterol; PAD, peripheral arterial disease; PCSK9, proprotein convertase subtilisin/kexin type 9.

# Table S2. Performance of different machine-learning algorithms in claims-based models

| **Model type** | **Accuracy score** | **Precision score** | **Recall score** | **F1 score** | **ROC AUC score** | **Average precision score** |
| --- | --- | --- | --- | --- | --- | --- |
| BDT | 0.9783 | 0.9655 | 0.918 | 0.9412 | 0.9845 | 0.9592 |
| FIGS | 0.9536 | 0.96 | 0.7869 | 0.8649 | 0.9606 | 0.9058 |
| LR L1 | 0.9505 | 0.8571 | 0.8852 | 0.871 | 0.972 | 0.948 |
| LR L2 | 0.9443 | 0.8413 | 0.8689 | 0.8548 | 0.9703 | 0.9451 |
| PU bagging FIGS | 0.9567 | 0.9273 | 0.8361 | 0.8793 | 0.9613 | 0.9236 |
| PU bagging RF | 0.9412 | 0.8387 | 0.8525 | 0.8455 | 0.9736 | 0.9212 |

BDT, boosted decision trees; F1, weighted average of precision and recall; FIGS, fast interpretable greedy-tree sums; LR, logistic regression; PU, positive unlabeled; RF, random forest; ROC AUC, area under the receiver operating characteristic curve.

# Table S3. Laboratory features explored for the FIGS Model 2

|  | True positive | Negative |
| --- | --- | --- |
| Patients, n | 141 | 29,713 |
| Patients with at least one LDL-C measurement >190 mg/dL after ezetimibe therapy, % | 28 | 0.2 |
| Patients with at least one LDL-C measurement >190 mg/dL after PCSK9 inhibitor therapy, % | 32 | 0.1 |
| Patients with at least two LDL-C measurements >190 mg/dL after ezetimibe therapy, % | 20 | 0.1 |
| Patients with at least two LDL-C measurements >190 mg/dL after PCSK9 inhibitor therapy, % | 18 | 0.0 |
| Maximum LDL-C value per patient, mean, mg/dL | 226.8 | 120.6 |
| Total LDL-C tests per patient, mean, n | 401 | 90 |
| LDL-C tests per day per patient, mean, n | 39 | 24 |
| Total LDL-C test days per patient, mean, n | 10 | 4 |

LDL-C, low-density lipoprotein cholesterol; PCSK9, proprotein convertase subtilisin/kexin type 9.
